# The influence of electrode array design, scalar dislocation and insertion technique on postoperative vertigo in CI surgery – a prospective study

**DOI:** 10.1007/s00405-024-09147-1

**Published:** 2024-12-12

**Authors:** Manuel Christoph Ketterer, A. K. Rauch, R. L. Beck, T. F. Jakob, L. Fries, A. Aschendorff, S. Arndt, F. Everad

**Affiliations:** 1 Department of Otorhinolaryngology, Medical Center, Faculty of Medicine, University of Freiburg, Freiburg, Germany; 2 Department of Otorhinolaryngology – Head and Neck Surgery, University of Freiburg, Killianstrasse 5, 79106 Freiburg, Germany

**Keywords:** Cochlear implant, Vertigo, Dislocation, Vestibular function

## Abstract

**Objectives:**

This study aimed to examine the effect of electrode array design, insertion angle, scalar position, and insertion technique on the occurrence of postoperative subjective vertigo following cochlear implant (CI) surgery using questionnaires in conjunction with objective vestibular functional measurements.

**Materials and methods:**

We prospectively evaluated subjective vertigo using the Dizziness Handicap Inventory (DHI). Additionally, we performed videonystagmography, video head-impulse tests, and vestibular-evoked myogenic potentials to assess the objective vestibular function preoperatively, at four weeks and 12 months after CI. These results were compared with those of postoperative imaging using digital volume tomography.

**Results:**

Postoperative vertigo was observed in 2 out of 62 patients (3%). Cochleostomy (*n* = 8) did not lead to an increase in postoperative vertigo. Functional diagnostics revealed abnormalities in up to 23% of patients without subjective dizziness. In our patient cohort, neither electrode array dislocation nor increasing insertion depth was associated with an increase in postoperative vertigo.

**Conclusion:**

Both postoperative vertigo occurrence and electrode array dislocation rates have significantly decreased due to the optimized atraumatic electrode array design and improved surgical insertion techniques. Neither dislocation nor cochleostomy appeared to induce vertigo but the sample size was too small to draw definitive conclusions.

## Introduction

Cochlear implant (CI) treatment is a well-established option for hearing rehabilitation in patients with bilateral deafness, single-sided deafness, and asymmetric hearing loss [1–4]. Many patients report experiencing acute dizziness after implantation [5–7]. The incidence of postoperative vertigo varies significantly among studies, ranging from 17.4% [7] to as high as 60% [5, 6, 8–10]. Up to a third of the CI patients complain of persistent dizziness [5, 8]. Basta et al. [5] observed that patients with CI who reported postoperative dizziness exhibited deficits in cervical vestibular-evoked myogenic potentials (cVEMPs), indicating a loss of saccular function. These deficits can be attributed to potential fibrosis in the vestibule [11] and damage to the saccular membrane caused by the insertion of the electrode array [5, 12, 13].

Several studies have examined the influence of the surgical approach on postoperative vertigo occurrence [7, 14, 15]. These studies report that a round window access seems to induce less postoperative dizziness than a cochleostomy, but these findings are based on small patient groups. In 2007, Aschendorff et al. [16] reported for the first time that postoperative speech perception depends on the scalar position and is significantly better with electrode arrays inserted into the scala tympani, a finding confirmed by subsequent studies [17, 18].

To date, there has been no work examining the influence of scalar position and dislocation of the electrode array on postoperative dizziness prospectively in a large study cohort. Additionally, there is still no systematic prospective study examining the influence of the electrode array design and scalar position on postoperative vertigo and functional deficits of the peripheral vestibular organ. The aim of this prospective study was to investigate the frequency, genesis, and duration of postoperative dizziness after CI. Furthermore, we aimed to examine the influence of the electrode array design on dislocation and traumatic insertion and possible vestibular dysfunctions due to structural injuries within the cochlea and vestibule. Moreover, the influence of the insertion technique (round window insertion versus cochleostomy) on postoperative vertigo was examined.

## Methods

**Fig. 1:**
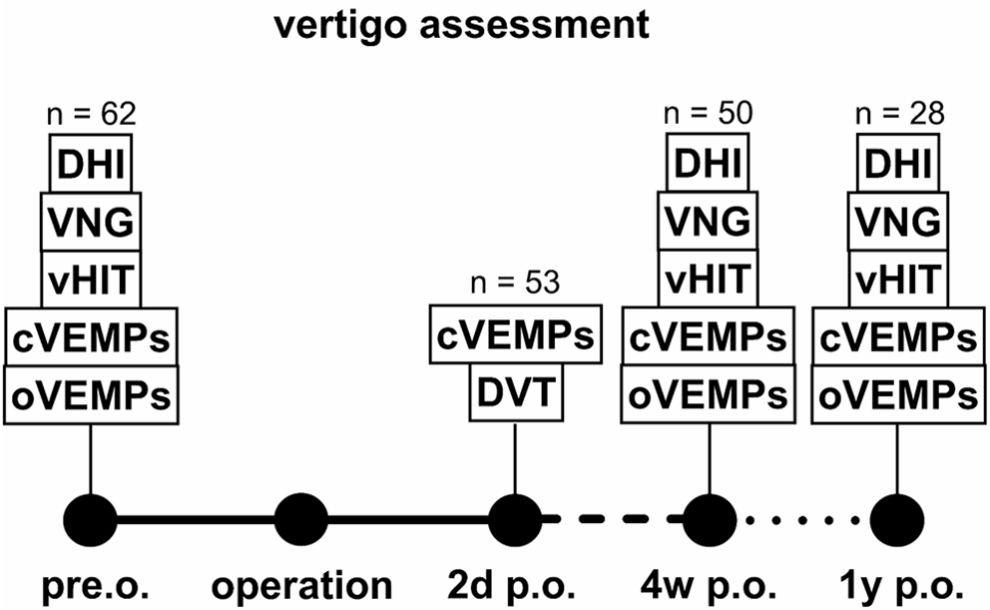
Study protocol with the testing battery preop., 2 days, 4 weeks and 1 year following surgery (VEMP = vestibular evoked myogenic potentials, HIT = video head-impulse test, VNG = videonystagmogram = caloric testing, DVT = digital volume tomography, DHI = dizziness handicap inventory)

Adult patients underwent regular CI pre-examination with objective measurements via videonystagmography (VNG), video head-impulse-test (vHIT), and cervical and ocular vestibular-evoked myogenic potentials (c and oVEMPs) (Fig. 1, study protocol). Subjective dizziness was evaluated with the Dizziness Handicap Inventory (DHI). Subsequently, patients underwent CI after detailed consultation at the Implant Center Freiburg, University hospital Freiburg, Germany, where they chose the manufacturer themselves. Depending on the surgical approach and manufacturer, the surgeon made the decision regarding whether to perform round window insertion or cochleostomy. Postoperatively, routine digital volume tomography (DVT) for position control, determination of scalar position, insertion angle, and dislocation determination were performed for all patients during the hospital stay (1–3 days after CI).

Two to three days after CI surgery cVEMP measurements were made (during the hospital stay) at various frequencies to assess direct postoperative saccular disturbances. At the initial adjustment (4–6 weeks after CI) of the implant, we performed cVEMPs and vHIT, and patients completed the DHI. One year after CI, we measured the utricular and saccular function (c and oVEMPs), vHIT and VNG, as well as assessment of subjective dizziness via DHI.

We performed statistical analysis using SPSS (SPSS Inc., Chicago, IL). P-values under 0.05 indicated statistical significance, and tables were designed in Microsoft Excel and Microsoft PowerPoint. This prospective study approved by the Hospital’s Ethics Committee in accordance with the Declaration of Helsinki (Washington, 2002) (Ethics Committee approval number: 129/19) and was registered in the German Clinical Trials Register (www.drks.de/ DRKS00034647).

## Results

The cohort consisted of a total of 62 patients (32 females and 30 males), aged from 22 to 85 years with a mean age of 58 years. Electrode arrays from all four available manufacturers: Cochlear™ (29), MED-EL (22), Advanced Bionics (AB) (9), and Oticon (2) were incorporated. Out of these 62, 38 were left ears and 24 were right ears.

A total of 28 patients exhibited bilateral CI indication and 3 patients already had implants on the contralateral side. Our cohort included 21 patients with asymmetric hearing loss and 13 with single-sided deafness. We identified recurrent sudden sensorineural hearing loss (SSNHL) in 17 patients, progressive hearing loss in 22 patients, and sudden deafness in 2 patients. Additionally, we observed one patient with an enlarged vestibular aqueduct, two with genetically inherited severe hearing impairment, two post-traumatic cases, and 16 with an unknown etiology. A total of 26 patients had preexisting pathological VNG results (> 25% asymmetry or < 12°/s speed of eye movement). These patients had the same etiology distribution as the whole study group. In total the study cohort had a normal mean vestibular function as shown in Table 1. The cohort included one patient with a vestibular schwannoma, who had undergone neurosurgical tumor resection before, and was, therefore excluded from further postoperative analysis. Furthermore, patients with otoclerosis and suspected endolymphatic hydrops have been excluded.

**Table 1:** Table demonstrating the mean preoperative vestibular function of the total study cohort measured by videonystagmography (VNG) and video head impuls test (vHIT)

Preoperative vestibular function	VNG: unilateral weakness [%]	VNG: Directional preponderance [%]	VNG: Total caloric response [°/s]	vHIT: Gain right lat.	vHIT: Gain left lat.	vHIT: Gain right post.	vHIT: Gain left post.	vHIT: Gain right ant.	vHIT: Gain left ant.
mean	32.1	19.3	41.2	1.0	0.9	1.0	0.9	0.9	0.9
standard deviation	28.5	20.6	22.6	0.2	0.2	0.4	0.3	0.3	0.3

Only 2 out of 62 patients (3%) reported postoperative vertigo two days after CI (see Table 2). Four weeks after CI, both patients exhibited conspicuous results in vHIT ipsilateral to the CI-supplied ear, each with overt saccades, which transitioned to covert saccades one year after CI, but still displayed a reduction in gain. C and oVEMPs were not remarkable at any of the measurement points for both patients. Both patients showed a decrease in subjective dizziness symptoms one year after CI. For the rest of the patients without subjective dizziness, abnormalities were still observed in up to 23% of the cases postoperatively (Table 3) during functional diagnostics. Table 3 shows the distribution of these abnormalities on the ipsilateral side, specifically the CI ear, immediately after CI, at four weeks, and one year postoperatively, in comparison to preoperative measurements of vHIT, cVEMPs, oVEMPs, and VNG. As recommended in previous studies, we evaluated the vHIT regarding its gain (< 0.7), covert and overt saccades, c and oVEMPs as pathological > 0.4, and the VNG with a side difference > 20%. The two sides were compared regarding their reaction to cold and warm water irrigation at 33 °C and 40 °C as recommended by multiple previous studies [7, 19–23]. Table 3 shows that up to 23% of the patients experienced functional vestibular deficits after CI, but without subjective complaints in the form of vertigo.

**Table 2:** Distribution table and functional diagnostics of the 2 patients with immediate postoperative vertigo (TD = dislocated out of scala tympani, RW = round window insertion, vHIT = ipsilateral video head impulse test to CI ear. ant = anterior, post = posterior, hor = horizontal: Gain and overt/ covert saccades)

Patient 1	Preop.	2 days *p*.o.	4 weeks *p*.o.	1 year *p*.o.
SPN	no	yes	no	no
DHI	0	/	0	0
cVEMPs (500)	0.35	0.05	0.06	0.19
cVEMPs (1000)	0	0.18	0.1	0.04
0VEMPs (500)	0.32	/	0.2	0.03
oVEMPs (1000)	0.07	/	0.04	0.19
vHIT ant	ok	/	ok	ok
vHIT post	ok	/	0.26 overt + covert s.	0.29 overt + covert s.
vHIT hor	ok	/	ok	ok
VNG	ok	/	/	ok
EA		Flex^28^ (MED EL)		
Coverage (°)		534.4		
Scalar position		TD		
Approach		RW		
Patient 2	Preop.	2 days p.o.	4 weeks p.o.	1 year p.o.
SPN	no	no	no	no
DHI	28	/	56	12
cVEMPs (500)	0.39	0	0.48	0.1
cVEMPs (1000)	0.29	0.17	0.16	0.07
oVEMPs (500)	0.27	/	/	0.25
oVEMPs (1000)	0.11	/	0.09	0.02
vHIT ant	ok	/	ok	ok
vHIT post	ok	/	ok	ok
vHIT hor	ok	/	0.26 overt s.	0.5 covert s.
VNG	ok	/	/	ok
EA		CI 622 (Cochlear™)		
Coverage (°)		375.3		
Scalar position		ST		
Approach		RW		

**Table 3:** Distribution table of functional diagnostics of the 60 patients without subjective vertigo symptoms (vHIT, c and oVEMPs and VNG: n with pathologic testing / n total / percentage)

	vHIT	cVEMPs 500 Hz	oVEMPs 500 Hz	VNG	DHI (mean)
preop	2/60 (3.3%)	8/56 (14.3%)	6/58 (10.3%)	10/57 (17.5%)	18.2
3 days	/	9/52 (17.3%)	/	/	/
4 weeks	2/54 (3.7%)	4/51 (7.8%)	3/51 (5.9%)	/	21.1
1 year	4/28 (14.3%)	2/28 (7.1%)	0/26 (0%)	6/26 (23%)	16.2

Electrode array portfolios from the four manufacturers (Table 4) were evaluated. Round window insertion was used in 53 out of 62 (85.4%) included ears. Additionally, only three electrode arrays exhibited dislocation from scala tympani into scala vestibuli in the postoperative DVT scans (Table 4). Two electrode arrays were inserted into the scala vestibuli after cochleostomy (Table 4). In one case, the preoperative MRI showed a low contrast agent uptake in the area of the scala tympani, which led to the cautious opening of the round window membrane. During this procedure, firm tissue was observed behind the round window membrane. After its removal, ossification of the scala tympani was detected. Initially, an attempt was made to drill out the bone of the scala tympani to achieve a scala tympani insertion, but this was unsuccessful. The opening was less than a millimeter wide and was visible at the end of the lower turn. An attempt was made to insert a test electrode, but this also failed. Therefore, the scala vestibuli was opened and found patent resulting in a successful insertion. In these included patients neither the surgical approach (cochleostomy versus round window insertion), scalar position nor increasing angular insertion depth were leading to an increased risk of postoperative vertigo.

**Table 4:** Numbers of included manufacturers and electrode arrays with their dislocation rates and the surgical approach as well as angle of array dislocation

Manufacturer	Electrode array	*n*	Scalar position [*n*]				Surgical approach [*n*]			Dislocation angle (°)
			ST	TD	SV	VD	RW	ERW	CS	
Cochlear™	CI612	1	0	1	0	0	0	0	1	180.7
Cochlear™	CI622	21	21	0	0	0	21	0	0	-
Cochlear™	CI632	7	7	0	0	0	7	0	0	-
MED EL	Flex^26^	15	15	0	0	0	12	0	3	-
MED EL	Flex^28^	7	5	1	1	0	6	0	1	apical
Advanced Bionics	HiFocus™ SlimJ	8	7	0	1	0	6	0	2	-
Advanced Bionics	HiFocus™ Mid-Scala	1	0	1	0	0	0	1	0	184.4
Oticon	EVO^®^	2	2	0	0	0	1	0	1	-

## Discussion

This prospective study examined the effect of the surgical approach, scalar position, electrode array design on postoperative vertigo. Two out of 62 patients (3%) had postoperative vertigo and demonstrated pathological results in vHIT as well as in the DHI. Previous studies within the last 20 years have reported varying incidences of postoperative vertigo after CI, ranging from 0.33 to 75% [12, 13, 24–30]. Hänsel et al. [7] reported a 17.4% incidence of new-onset vertigo in 303 out of 1743 patients included in 31 studies and of persistent vertigo complaints in 7.2%. Several studies reported morphological changes in both inner ear structures after CI surgery, specifically in the cochlea and vestibulum [25, 31]. Hänsel et al. [7] hypothesized that postoperative vertigo after CI occurs multifactorial due to damage to the organ of Corti and labyrinth structures caused by mechanical damage during surgery, receptor alteration, and/or effects leading to diminished central nervous system function [32–34].

Matin et al. [24] reported 12 out of 71 patients with postoperative vertigo. Six of them (8.4%) had new postoperative vertigo, and six had preoperative gait unsteadiness. Nevertheless, they only performed caloric testing (VNG) and clinical HIT, but not vHIT. Additionally, two out of the 12 described vertigo patients showed intraoperative abnormalities. Matin et al. [24] reported dislocation rates of 16.9%, and that the perimodiolar electrode arrays seemingly induced less vertigo than the lateral wall electrode arrays.

The two patients included in our study had pathological vHIT results and subjectively described vertigo two days after CI and four weeks after CI, but also reported a significant improvement of symptoms one year postoperatively. In this study, one patient was inserted with a CI622 and the other one with a Flex28. Our findings do not confirm the findings of Basta et al. [5], who reported that CI patients who complained of dizziness postoperatively showed deficits in cVEMPs. Furthermore, we could not identify the perimodiolar electrode array design leading to higher incidences of postoperative vertigo. However, the rate of 3% is very low, and we are therefore cautious with definitive conclusions in this regard. Our findings are in line with those of Matin et al. [24] who reported that impaired vestibular function did not always correlate with subjective vertigo perception. As shown in Table 3, postoperative abnormalities were mainly found in VNG, vHIT, and cVEMPs in up to 23% of the patients who did not subjectively complain of dizziness. Hänsel et al. [7] reported a reduction in saccular function in 13 out of 116 studies included in their meta-analysis. However, because Abouzayd et al. [35] described difficulties in choosing the correct vestibular test battery due to low sensitivity (21% for VNG, 32% for cVEMPs, and 50% for HIT), we highly recommend performing the complete vestibular test battery to have clear diagnostic accuracy regarding vestibular function, especially for future procedures, such as reimplantation.

Because both patients with vertigo included in this study were inserted via round window insertion, we could not test the hypothesis that cochleostomy leads to high vertigo risks. Hänsel et al. [7] reported higher vertigo rates in cochleostomy patients than in round window inserted ones. Nevertheless, they reported similar results for nystagmography and cVEMPs for round window insertion and cochleostomy. However, Rah et al. [36] reported no vertigo in round window inserted patients.

In this study, we aimed to investigate whether variations in electrode array design and scalar position influence the occurrence of postoperative vertigo and vestibular dysfunction. Among the 62 patients we examined, only 3 (4.8%) exhibited dislocated electrode arrays. Specifically, one case involved the Contour Advance of CochlearTM, one case the Flex28 of MED-EL, and one case the MS of AB. Additionally, only two electrode arrays were inserted into the scala vestibuli, comprising one Flex28 by MED-EL and one SlimJ by AB.

Considering the dislocation rates reported in previously, there has been a significant decrease in the risk of electrode array dislocation most probably due to the optimized atraumatic electrode array design and enhanced surgical techniques. Aschendorff et al. [16] reported dislocation rates of up to 28.5%, Ketterer et al. [37] reported 21.6%, and Ketterer et al. [38] reported 7.8%. In this study, we did not observe incidences of dislocation or scala vestibuli insertions influencing subjective vertigo or objectively measurable disturbances in vestibular function testing. However, the number of dislocations and scala vestibuli insertions were too small to draw definitive conclusions.

Future multicenter studies should explore whether the risks of dislocation and postoperative vertigo depend on the electrode array design. Such studies would be crucial in identifying factors that determine the preoperative selection of electrode arrays and surgical approach.

## Conclusion

Our findings demonstrate that advancements in electrode array design and optimized surgical techniques have significantly reduced the incidence of postoperative vertigo, which was observed in only 3% of patients, compared to higher rates reported in earlier studies. Neither scalar dislocation nor the cochleostomy approach appeared to increase the risk of vertigo; however, the limited sample size prevents definitive conclusions. Notably, vestibular function abnormalities were identified in up to 23% of patients who reported no subjective dizziness, highlighting the need for comprehensive vestibular evaluations.
